# Monitoring Neuromuscular Activity during Exercise: A New Approach to Assessing Attentional Focus Based on a Multitasking and Multiclassification Network and an EMG Fitness Shirt

**DOI:** 10.3390/bios13010061

**Published:** 2022-12-30

**Authors:** Aslan B. Wong, Diannan Chen, Xia Chen, Kaishun Wu

**Affiliations:** 1 College of Computer Science and Software Engineering, Shenzhen University, Shenzhen 518061, China; 2 Department of Psychology, University of Wisconsin-Milwaukee, Milwaukee, WI 53211, USA; 3 Information Hub, The Hong Kong University of Science and Technology (Guangzhou), Guangzhou 511453, China

**Keywords:** wearable device, biosignal sensing, exercise monitoring, attentional focus, neuron network

## Abstract

Strengthening muscles can reduce body fat, increase lean muscle mass, maintain independence while aging, manage chronic conditions, and improve balance, reducing the risk of falling. The most critical factor inducing effectiveness in strength training is neuromuscular connection by adopting attentional focus during training. However, this is troublesome for end users since numerous fitness tracking devices or applications do not provide the ability to track the effectiveness of users’ workout at the neuromuscular level. A practical approach for detecting attentional focus by assessing neuromuscular activity through biosignals has not been adequately evaluated. The challenging task to make the idea work in a real-world scenario is to minimize the cost and size of the clinical device and use a recognition system for muscle contraction to ensure a good user experience. We then introduce a multitasking and multiclassification network and an EMG shirt attached with noninvasive sensing electrodes that firmly fit to the body’s surface, measuring neuron muscle activity during exercise. Our study exposes subjects to standard free-weight exercises focusing on isolated and compound muscle on the upper limb. The results of the experiment show a 94.79% average precision at different maximum forces of attentional focus conditions. Furthermore, the proposed system can perform at different lifting weights of 67% and 85% of a person’s 1RM to recognize individual exercise effectiveness at the muscular level, proving that adopting attentional focus with low-intensity exercise can activate more upper-limb muscle contraction.

## 1. Introduction

Strength training has been the most fascinating exercise of the last decade. Strength training is used by default in workouts to build strength, muscle mass, and joint strength. However, everyone has different fitness goals. Long-term outcomes vary depending on the exercise routine; however, everyone wants to train efficiently every time. Depending on the program, exercise objectives have several definitions. Endurance, hypertrophy, maximum strength, and power are the four distinct goals outlined by the National Strength and Conditioning Association (NSCA) [1]. For each plan, the weight and repetition count must be changed. There are numerous possible lifts under these circumstances, depending on the muscles needed, the equipment used, and the speed, duration, and intricacy of the movements. Put simply, training can be more successful if people focus on the use and movements of their muscles during exercise. Conscious awareness of muscles and movement during a full range of motion can increase muscle fiber activation, which is known as attentional focus [2,3,4,5,6].

The COVID-19 pandemic has resulted in social distancing measures and led to a “new normal” exercise practice. Instead of going to the gym, training at home became normal. In the field of fitness training, weightlifting equipment is used everywhere, but strength training does not take up space, and a dumbbell can be used to perform a variety of exercises. Emerging online platforms, including APPs, can also be used to guide and monitor practitioners. However, many people do not monitor their exercise performance on a variety of platforms because they are occasionally misaligned with their objectives, and there is no practical solution to give users feedback on training effects related to attentional focus at the muscle level [7,8,9].

The concept of attentional focus has important implications for fitness training. Sports science research refers to attentional focus as an individual focusing on muscles and movements while performing a given movement or activity [6]. For example, when performing a bicep curl, attentional focus may be directed at “stress the biceps” [10]. In this context, attentional focus is conscious and deliberate regarding muscle contraction. We can focus on the tension created during exercise in a specific voluntary muscle, distinguishing between the passive and active movements of the weight. Several studies in [11,12] revealed that muscle activation is more remarkable when subjects are instructed to focus their attention on the targeted muscle region. In [13], subjects could significantly increase normalized EMG (electromyography) activity by focusing on the pectoralis or triceps on the respective muscles while performing curls. These results are consistent with [14], in which it was mentioned that attentional focus can increase muscle activity or contraction. Similarly, research has shown that the adoption of attentional focus on the target muscle can result in higher activation of the pectoralis major, biceps brachii, and triceps brachii [11]. The effect of voluntary motor effort during a low-intensity muscle exercise training program on increasing muscle strength for six weeks has been investigated [15]. The results suggest that the level of effort involved in resistance exercise training plays a critical role in determining strength increase. Low-intensity muscle exercise with attentional focus or high mental effort can lead to increased strength. This suggestion is consistent with the National Strength and Conditioning Association (NSCA) and the American College of Sports Medicine (ACE), both of which state that hypertrophy or increasing muscle fiber training corresponds to a resistance of approximately 67% of a person’s 1RM (one-repetition maximum) for a good balance of strength and muscular conditioning [1,16,17].

In [18], the authors mentioned that the majority of people do not realize that the Central Nervous System (CNS) is an essential factor in maximizing muscle strength, as muscle contraction begins with an impulse from the brain. The misconception of “no pain, no gain” is that lifting heavier weights could result in stronger muscles [19]. Training with attentional focus results in greater muscle strength during maximal voluntary contraction [4,15]. Based on the neuromuscular connection mentioned above, no available method could assist practitioners with attentional focus during strength training. It would be much better if we knew how exercise results from attentional focus each session. However, numerous studies have been conducted on wearable devices for fitness and exercise that are motivated by user activities related to achieving adequate performance or exercise effectiveness during training, such as posture and form [20,21], muscle fatigue [8,22,23], and fitness tracking [24,25]. In a practical scenario, the primary challenge is to develop a recognition model based on different exercises, weight-lifting, attentional focus, and users.

The neuromuscular system comprises motor neurons, sensory neurons, and muscle fibers. Body movement, posture control, and breathing are also controlled by this system. In physical exercises that involve neuromuscular control, the body can produce forces as well as stabilize and reduce external forces dynamically through movement, which in essence keeps the body balanced. Many researchers have studied brainwaves and muscle tasks using EEG signals to map dynamic sources of cortical signals [26,27]. However, weight-lifting exercises require intense movement, resulting in high artifacts. We will use EMG sensors instead of EEG sensors to investigate the connection with upper-limb muscle function.

**Figure 1:**
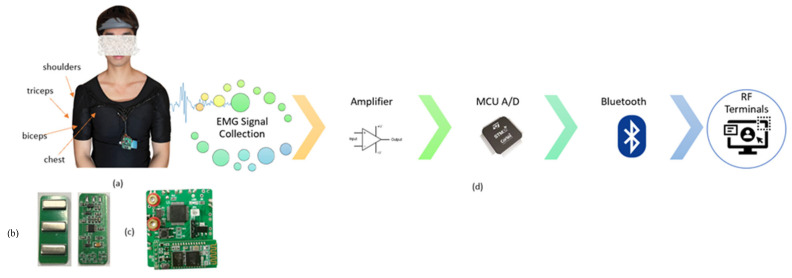
(**a**) Positions of EMG sensing electrode in wearable EMG fitness shirt; (**b**) EMG sensing electrode; (**c**) PCB; (**d**) Signal transmission pipeline.

**Figure 2:**
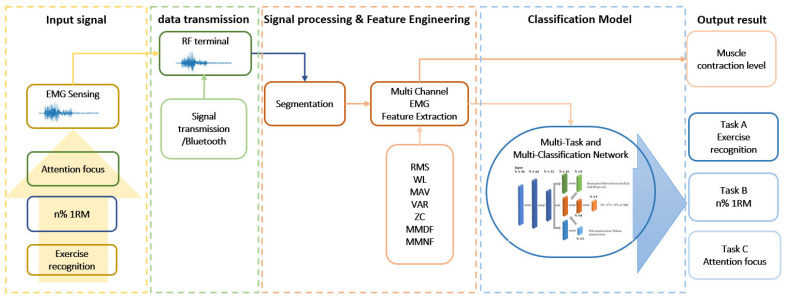
System framework.

To date, we have acknowledged that attentional focus is an essential element of sports and exercise performance in hundreds of studies [11,12,28]. Research on attentional focus in various muscle groups has not been adequately conducted [9,11]. The difficulty lies in making the concept of attentional focus useful in a real-world situation. It is challenging to reduce the clinical measurement of wearable devices [29]. In addition to the size and hardware performance, user comfort is a trade-off. For end users, there is still no system available to track attentional focus in practical settings. To overcome this challenge, we developed a wearable device to empirically investigate attentional focus, which results in muscle contraction in various exercises in a practical scenario. We present a system to sense muscle activation signals and evaluate user performance in terms of attentional focus during strength training. Our custom design of a low-cost, dry, noninvasive electromyography or EMG sensing electrode that is highly conductive is designed with compactness and attachability to the wearable device in the form of an EMG fitness shirt, as shown in Figure 1. In this study, EMG signals were analyzed using a test involving five standard isolated and compounded muscles during free-weight exercises. To better assist people in exercising, we present a multitask and multiclassification network that treats different exercises (task A), attentional focus (task B), and different 1RM (task C) as three subtasks and classifies them uniformly. The system framework shown in Figure 2 was trained to detect the attentional focus condition that results in muscle contraction. The experimental results suggest that the proposed system can be used to monitor attentional focus through muscle contractions. To increase the number of muscle fibers or induce hypertrophy, this study also evaluated the efficacy of various strength training exercises. In addition, we discuss whether lifting lightweight objects is necessary to achieve the best results in terms of workout effectiveness. The contributions of this study can be summarized as follows:

- We develop a system with a wearable, eight-channel, noninvasive EMG fitness shirt to assist in sensing attentional focus during exercise.
- We develop a system comprising a multitask and multiclassification network to detect attentional focus on muscle contraction from EMG signals for tracking personal fitness at the muscular level.
- We implement and evaluate the system for attentional focus and muscle contraction at different lifting weights based on five standard exercises of isolated and compounded muscles.

We begin the rest of the paper with a discussion on related works of attentional focus, followed by experiments and system design. Finally, we discuss the results and evaluate this new strength-training tracking method.

## 2. Materials and Methods

### 2.1. Hardware Setup and System Framework

We designed a fitness-training shirt integrated with EMG sensors to offer reliable signal recording, as shown in Figure 1. The EMG shirt was integrated with an 8-channel EMG sensor. Eight EMG sensors were placed in the main upper-limb muscle groups: (1) two on the chest, (2) two on the shoulders, (3) two on the triceps, and (4) two on the biceps. An integrated wireless transmission terminal circuit is placed on the shirt. The EMG signal collected by dry electrodes passed through an analog front-end, a band-pass hardware filter, a signal amplifier, and a 512 Hz A/D converter, as shown in Figure 1, which reliably records the EMG signal with a 700 gain and 1000 Hz sampling rate and is transferred by a microprocessor (STM32F103RCT6, 32-bit ARM Cortex-M4) and Bluetooth serial module HC-05.

The system framework is illustrated in Figure 2. It starts by detecting attentional focus via a fitness shirt fitted with noninvasive dry electrodes that are firmly attached to the body and then simultaneously measures muscle activity during training. During standard lifting exercises, multitask and multiclassification networks are used to track the user’s attention during isolated and compound muscles in the upper limbs. Note that data processing was conducted offline following the collection of the experimental data.

**Figure 3:**
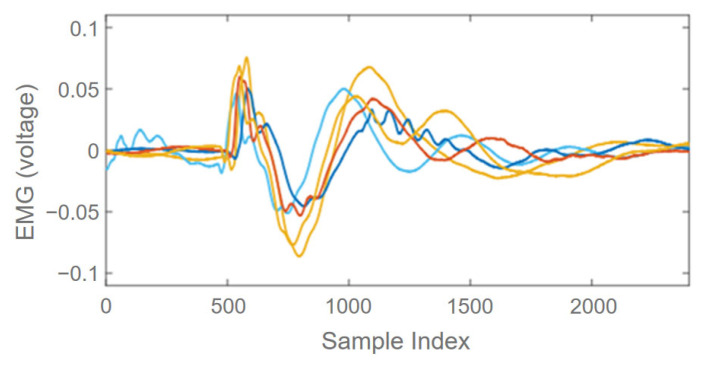
Raw EMG signals from five subjects performing the exercise in the pre-experiment.

Signal collection was based on the use of electrodes on the training muscle as the source and reference electrodes. We investigated the signal when the user wore the shirt because the device’s position and orientation might not be identical if the gesture signal is sensitive to this position/orientation; therefore, the EMG might not be viable for practical applications. In this pre-experiment, five people were asked to wear the device and perform a bench press, producing natural diversity across the subjects. Figure 3 shows the consistency of the detected signal when the same exercise gesture was performed by the subjects. The raw measurements were reasonably consistent with the specific exercises. The recognition performance is discussed in Section 3.2.

### 2.2. Experimental Setup

**Figure 4:**
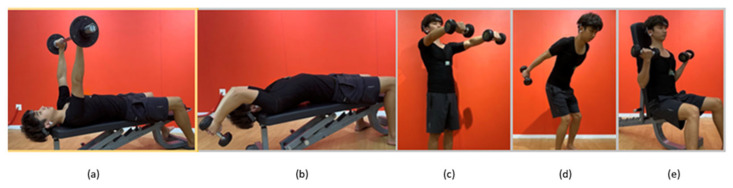
Exercise and its muscles: (**a**) Bench press: pectoralis major, lateral deltoids, triceps; (**b**) Pullover: pectoralis major (chest); (**c**) Front rise: lateral deltoids (shoulders); (**d**) Kick back: triceps brachii; (**e**) Bicep curls: biceps.

We recruited 12 healthy male college students who participated in personal weight training activities (12 subjects, age (M = 23.92 years, SD = 1.93 years), height (M = 175.17 cm, SD = 4.72 cm), weight (M = 72.89 kg, SD = 5.75 kg), and BMI (body mass index) (M = 23.81, SD = 2.33). To enable us to determine the force, participants underwent a strength assessment. We targeted five exercises recommended by the resistance training guide for healthy adults [1]. The four isolated exercises were lying pullovers, front raises, kickbacks, and biceps curls, and the one compounded exercise was a bench press, as shown in Figure 4. These are among the most common exercises that target different muscle groups in the body with respect to the target muscles. Experiments align with constrained action as a research experiment with attentional focus for resistant training and weightlifting [15]. The subjects performed five exercises with dumbbells weighted with a mass equivalent to the estimated 67% and 85% of 1RM [16]. Each participant performed 12 repetitions of each exercise in each session, with weights considered appropriate for their strength training. In total, each participant performed 72 repetitions of each exercise, resulting in 360 repetitions in the attention condition and without attentional focus. Reminders on the attentional focus (with and without) were given before each session. They were given rest periods of approximately 5 min between each session and condition. Data were collected at a fitness center on a university campus. Throughout data collection, subjects were instructed to perform five exercises following the exercise’s explicit instructions and performed without carrying weight for the baseline data. To capture intra-subject variability, each participant attended six sessions of data collection on three days.

Procedure:

1. Instruct the user to perform five exercises in the following order: Bench Press, Bicep Curl, Triceps Kickback, Front Raise, and Lying Pullovers;
2. Ask the user to prepare for 5 min before start;
3. Start session 1: 0% weight, 12 repetitions without attentional focus;
4. Rest for 5 min;
5. Start session 2: 0% weight, 12 repetitions with attentional focus;
6. Rest for 5 min;
7. Start session 3: 67% 1RM, 12 repetitions without attentional focus;
8. Rest for 5 min;
9. Start session 4: 67% 1RM, 12 repetitions with attentional focus;
10. Rest for 5 min;
11. Start session 5: 85% 1RM, 12 repetitions without attentional focus;
12. Rest for 5 min;
13. Start session 6: 85% 1RM, 12 repetitions with attentional focus;
14. Rest for 5 min;
15. Start a new exercise and repeat from Step 3;
16. When the user completes the five exercises, the user is asked to rest.

### 2.3. Segmentation

Our 8-channel EMG sensor was limited to 0–4096. When collecting the data, we normalized the data for each channel to a range of 0–1, with a minimum value of 0 and a maximum value of 4096. Then, we averaged the data from the eight channels for signal segmentation. First, we cut the average continuous EMG signal to obtain a single signal for each movement. Specifically, we performed high-pass filtering (cut-off frequency of 1 Hz) on the signal to remove the DC component. Next, we take 0.25 s as the frame length and 0.1 s as the frame step to calculate the frame energy. We set the signal threshold as *ξ*. When the frame energy exceeded ξ, it was recorded as the start frame, *f_t_*. When the frame energy is lower than ξ, it is recorded as the end frame *f_e_*. We removed signals with durations of less than 1.5 s or for which the maximum energy was lower than the threshold δ, and then obtained the corresponding starting point *t_s_* and ending point *t_e_* (*t_s_* = *f_s_* × frame step; *t_e_* = *f_e_* × frame step) of the original signal. We extended the signal forward and backward for 0.15 s (30 sampling points), respectively, to obtain a complete signal and finally obtain the 8-channel data of the EMG signal in a single movement for feature extraction.

### 2.4. Muscle Contraction Measurement

The typical method to develop muscle strength is to contract the muscle to its maximum potential at any given load. In strength training, 1RM is the maximum number of repetitions that can be achieved with a given resistance or weight. Here, we asked subjects to exercise with dumbbells weighted to an estimated 67% and 85% of 1RM with and without attentional focus, respectively. The goal of estimating muscle contraction is to track the effectiveness of attentional focus during exercise. If muscle contraction with attentional focus is greater than that without attentional focus, it is interpreted that the targeted muscle is activated with respect to the minimum of the maximal low-intensity voluntary contraction [11,15]. The root mean square (RMS) value was used to measure the muscle activation level. We normalized the data of each channel to the range of 0–1 with a minimum value of 0 and a maximum value of 4096; averaged the data of eight channels; performed high-pass filtering (cut-off frequency of 1 Hz) to remove the DC component; and calculated its RMS. The RMS is modeled as an amplitude-modulated Gaussian random process, whose RMS is related to the constant force and muscle contraction. The RMS calculation is as follows:

(1) $$\mathrm{RMS}=\sqrt{\frac{1}{N}\sum_{i=1}^{N}x_{i}^{2}}$$

### 2.5. Feature Extraction

Seven features were selected from EMG signals [8,29,30,31]: (1) The Root Mean Square (RMS) is modeled as an amplitude-modulated Gaussian random process, whose RMS is related to the constant force and non-fatigue contraction. (2) The waveform length (WL) is the cumulative length of the waveform over the time segment. The WL is related to the amplitude, frequency, and time of the waveform. (3) The mean absolute value (MAV) is similar to the average rectified value (ARV). It can be calculated using the moving average of a full-wave rectified EMG signal. In other words, it was calculated by taking the average of the absolute value of the EMG signal. This is an easy way to detect muscle contraction levels and is used in myoelectric control applications. (4) Variance of the EMG (VAR) uses the power of the EMG signal as a characteristic. Generally, variance is the mean value of the square of the deviation of that variable. However, the mean EMG signal is close to zero. (5) Zero crossing (ZC) is the number of times the amplitude of the EMG signal crosses the zero y-axes. The EMG feature uses a threshold condition to abstain from background noise. This feature provides an approximate estimate of frequency-domain properties. (6) The Modified Median Frequency (MMDF) occurs when the spectrum is divided into two regions with equal amplitudes. (7) The modified mean frequency (MMNF) is the average frequency. The MMNF is calculated as the sum of the amplitude spectrum product and the frequency divided by the total sum of the spectrum intensity.

### 2.6. Multitask and Multiclassification Network for Attentional Focus Exercise

**Figure 5:**
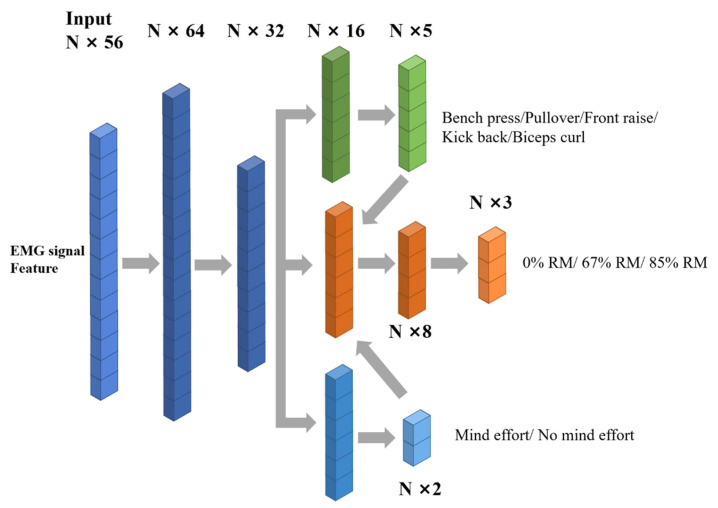
Multitask and Multiclassification Networks.

In this work, we propose a multitask and multiclassification network that considers different exercises (task A), attentional focus (task B), and 1RM (task C) as three subtasks and classifies them uniformly to better guide people during exercise. The proposed network architecture is shown in Figure 5. The input of the network consists of 56 features extracted from the EMG signal (eight channels, seven features for each channel). The three subtasks share the same input, and the features of the three subtasks are extracted from the input data through two fully connected layers (followed by a dropout layer). The unique features were extracted and classified through the full connection layers of the branches of the three subtasks. Subtask A classified different exercises, subtask B determined whether the exercise had a focus of attention, and subtask C determines different 1RMs. Multitask and multiclassification processes consist of three subtasks that share the same inputs. Twelve repetitions of each session for each person were divided into a training set, verification set, and test set for eight repetitions, two repetitions, and two repetitions, respectively, in which the shared features were extracted and the unique features were extracted for completion of the subtasks. All of the above full connection layers go through a sigmoid activation function. Task B, the classification of RM, did not parallel the other two tasks. Its classification depends on specific exercises and attentional focus because, for different exercises, different RMs may generate EMG signals with similar energy. For example, 67% RM for exercise 1 and 85% RM for exercise 2 may generate EMG signals with similar energy. Therefore, we designed a fusion layer before the classification layer of task B which combines the output vectors of tasks A and C with the feature vectors of task B before classification. Through the fusion layer, the exercise information and focus information are introduced into task B so that it can narrow the range of the feature space and obtain the correct RM.

Because each person’s physical function and muscle level were different, we trained a model for each subject. Each person’s data were divided into a 70% training set, 15% verification set, and 15% test set. In the training process, all these subtasks contribute to the overall loss function so that their performance can be considered. We define a loss function for the output vector of each subtask and then define the overall loss function as the (weighted) sum of these subtask loss functions. The loss function is defined as follows:

(2) $$ℒ\left(X,Y,\bar{Y}\right)=ℒ\left(X,\left(Y_{A},Y_{B},Y_{C}\right),\left({\bar{Y}}_{A},{\bar{Y}}_{B},{\bar{Y}}_{C}\right)\right)=\sum_{t\in T}\omega_{t}{ℒ}_{t}\left(X,Y_{t},{\bar{Y}}_{t}\right)$$

where *A*, *B*, and *C* represent the three subtasks, *X* is the input, *Y* is the real label, $\bar{Y}$ is the predicted output, and *t* is the task space {A, B, C}.

Because the subtasks in this study were supervised multiclassification tasks, the component loss function of each task was categorical cross-entropy. By defining loss as a linear combination of component losses, we obtain a set of super parameters *ω_t_* that can measure the impact of the component loss function, which can limit the impact of the tasks. Therefore, if the output of only one task is important for the application, it can be prioritized by assigning it a greater weight. In our experiment, these three tasks were equally important. Therefore, its weight was 1.

## 3. Results and Discussion

### 3.1. Evaluation Matric

The results of the experiment on practical scenarios are presented in the next section. Based on the evaluation, we divided the experiment into two categories: the robustness of the system and its impact on the user. A robust system analyzes how the system performs in practical scenarios on exercise recognition and attentional focus, whereas an impact system examines the relationship between a user’s attentional focus and the level of muscle contraction. To evaluate the robustness of the system for the recognition of attentional focus, we used precision (also called the positive predictive value) and specificity (also called the true negative rate) as criteria for recognizing the correct attentional focus performance. In this context, precision refers to the total number of positives of the actual attentional focus covered while exercising (correct identification). The performance of recognized exercises without attention was measured by specificity. For exercise and 1RM recognition, accuracy was used to measure the proportion of both true positives and true negatives. Using RMS for muscle contraction level, the performance of subjects was determined based on their muscle contraction level in relation to their attentional focus in different 1RMs.

### 3.2. Performance on Exercise Recognition (Task A)

**Figure 6:**
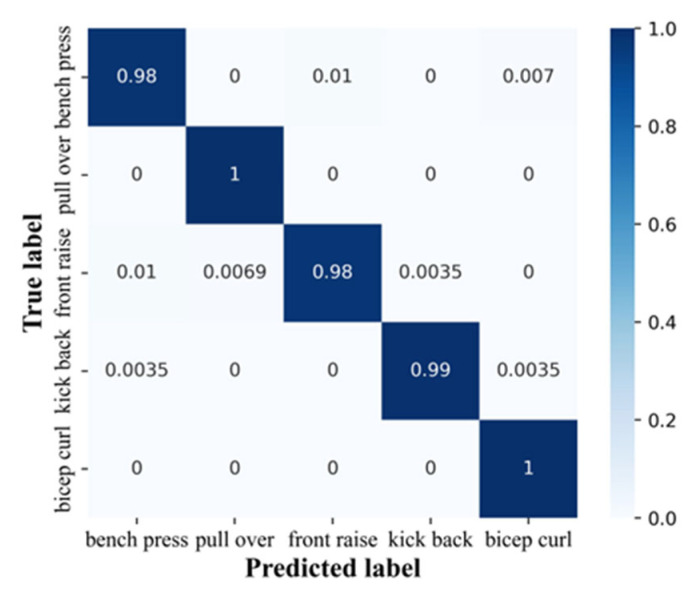
Performance in exercise recognition.

We further investigated the results of exercise recognition in detail. If the position and orientation of the device are not identical and the posture signal is sensitive to this difference, the EMG may not be suitable for practical applications. The raw data are still reasonably consistent with the exercise in question. As shown in Figure 6 and Table 1, two out of the five exercises achieved 100% accuracy and specificity. The EMG fitness shirt can achieve a high recognition performance accuracy of 99% across all five exercises involving the targeted muscles in a subject-exercise manner. Taken together, our results confirm that the system can achieve high recognition performance across all five exercises in a subject-exercise manner with respect to the targeted muscles. This experiment proves the consistency of the signal generated from the natural variation among subjects, demonstrating that the detected signals are consistent when subjects perform the same exercise posture.

### 3.3. Performance on User Attentional Focus Recognition (Task B)

**Figure 7:**
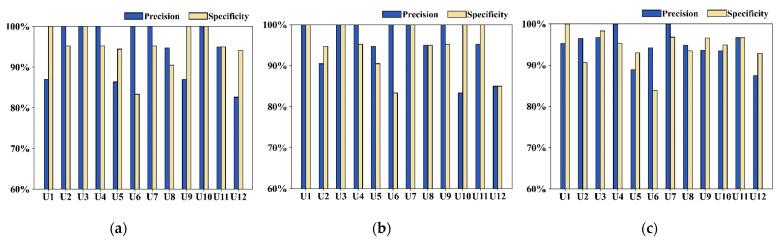
Performance in user attentional focus recognition at different 1RMs: (**a**) attentional focus recognition without carrying dumbbells; (**b**) attentional focus recognition lifting weight at 67% of the 1RM; and (**c**) attentional focus recognition lifting weight at 85% of the 1RM.

We first show the performance of the system through the recognition of attentional focus at different 1RMs. Figure 7 shows the results of the overall recognition performance for attentional focus at 67% and 85% of the 1RM and without lifting any weights. The most frequent classification output was selected as the result for each subject. Figure 7a shows the recognition performance of the user’s attentional focus without lifting any weight. Eight subjects had attentional focus recognition with greater than 90% precision, while the remaining four had more than 80% precision. As can be seen for the 67% 1RM in Figure 7b, the system achieved am attentional focus recognition precision greater than 90% for 10 subjects, while the remaining two achieved more than 80%. As shown in Figure 7c, the system also achieved an attentional focus recognition precision of 90% for 10 subjects at 85% of the 1RM, while the remaining two achieved more than 85%. To summarize the overall performance of the system in the recognition of attentional focus at 67% and 85% of the 1RM, good precision (M = 94.79%, SD = 3.77%) and specificity (M = 94.35%, SD = 4.20%) were achieved.

### 3.4. Performance on Different 1RM Recognition (Task C)

**Figure 8:**
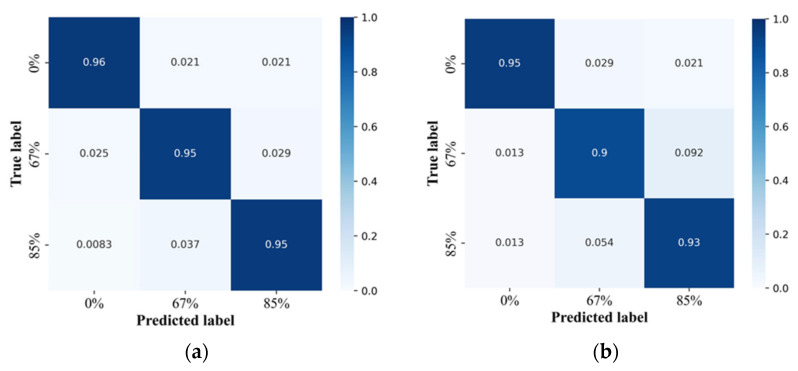
1RM Recognition Performance (**a**) with attentional focus and (**b**) without attentional focus.

As shown in Figure 8, our system can differentiate between 1RM recognition based on varying subjects, weights lifted, exercise type, and attentional focus. Our system achieved more than 90% accuracy in every 1RM, both with attentional focus (Figure 8a) and without attentional focus (Figure 8b). It seemed to have a slightly lower performance in the recognition of exercise without attentional focus, but it did not have a significant effect on the other recognition tasks, that is, attentional focus and exercise recognition. To summarize, the overall recognition performance of exercise with attentional focus at 67% and 85% of the 1RM was 95.33% accurate, and the recognition of exercise without attentional focus was 92.67% accurate.

### 3.5. Comparisons to Different Classification Models

**Figure 9:**
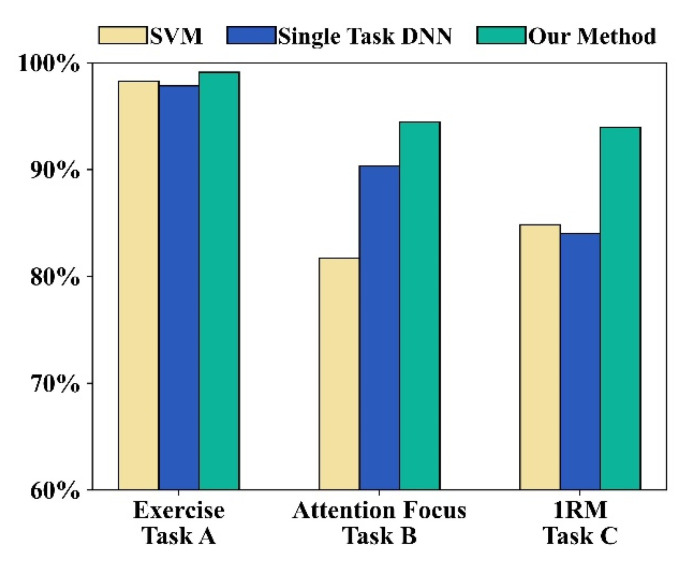
Comparison of recognition performance of different classification models.

We used a support vector machine (SVM), a deep neural network (DNN) of a single task, and our multi-task and multi-classification network to compare the overall system performance. We found the best hyperparameter of all the models to evaluate the performance of the learning algorithms, and we evaluated the detection performance for each model. For our multitasking and multiclassification network, we used the Adam optimizer with a learning rate of 0.001. For the DNN of a single task, the three branches of the proposed network were trained independently. The 1RM recognition network lost its fusion layer, and other network structures and settings remained unchanged. For SVM, we used RBF as the kernel function and the one-versus-one method to design a multiclassification SVM. The results are shown in Figure 9, which shows that our multitask and multiclassification network method can achieve the highest accuracies in the three recognition tasks. The SVM and DNN of the single task performed well in the exercise recognition task, but did not perform well in the attentional focus and 1RM recognition tasks. As previously mentioned, 1RM recognition depends on specific exercises and attentional focus. An SVM and a single-task network cannot provide such information. Furthermore, we should focus on analyzing the signals obtained during muscle contraction, which has been confirmed with single-task DNN or multitask and multiclassification networks.

### 3.6. Impact of Attentional Focus on User Muscle Contraction

**Figure 10:**
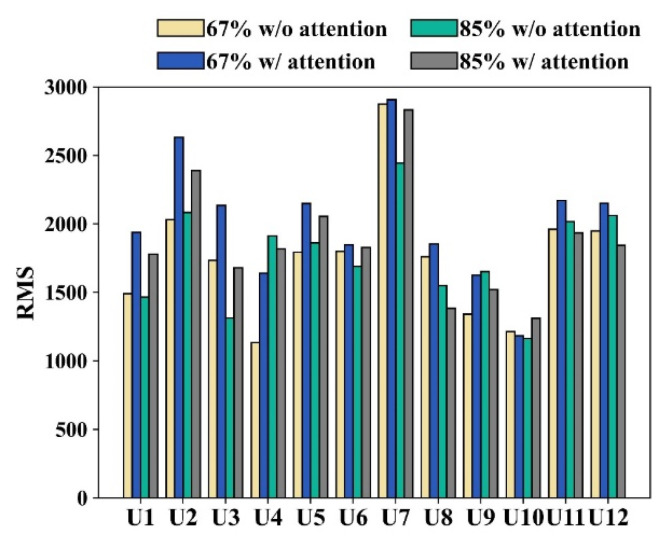
The results of muscle contraction of each subject were based on different attentional focus conditions and different lifting weights of the 1RMs.

The results in Figure 10 indicate the RMS of each muscle contraction under different conditions of attentional focus and at 67% and 85% of the 1RM, respectively. As mentioned earlier, our objective is exercise effectiveness of muscle strength, which can be achieved by low-intensity exercise according to the ACE’s recommendations of 67% and 85% of the 1RM. We then examined the muscle contraction to provide a detailed examination of the effectiveness of each subject’s exercise. A significant difference was found between exercise with and without attentional focus, which means that exercise with attentional focus has a high impact on muscle contraction at 67% and 85% of the 1RM. The results in Figure 10 also indicate that 10 of the 12 subjects had more muscle contraction during low-intensity weightlifting at 67% of the 1RM, which means that training with attentional focus can affect muscle strength [6,13,14]. This result is also valid for the goal of hypertrophy training, in which adopting attentional focus with lightweight lifting can activate more muscle contraction [1,17].

### 3.7. Impact of Attentional Focus on Muscle Contraction of Each Exercise

**Figure 11:**
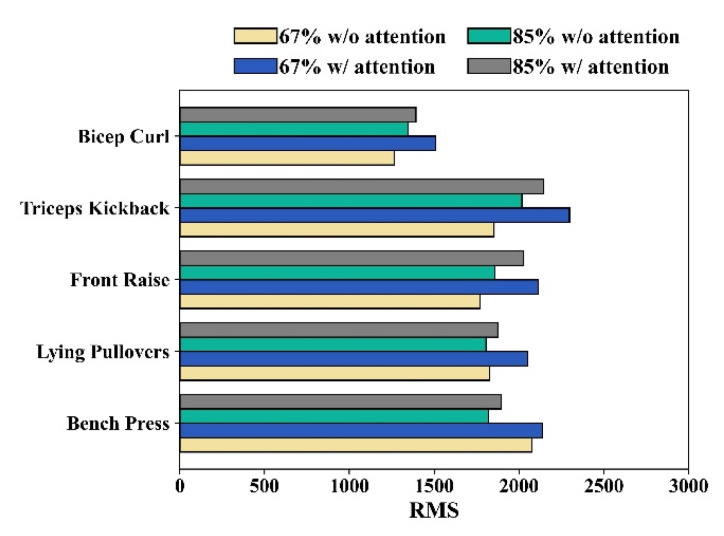
The result of muscle contraction for each exercise in a different attentional focus condition and different lifting weights of 1RMs.

In addition, we evaluated attentional focus under two conditions to determine the difference in muscle contraction. In this evaluation, we compared muscle contraction during each exercise. Figure 11 compares the muscle contraction between 67% and 85% RM. This result also responds to the hypertrophy training goal that adopts attentional focus through lightweight lifting, which can activate more muscle contraction. All exercises at 67% of the 1RM (pullover) had a more significant contraction than at 85% of the 1RM. Figure 11 also shows that exercises at 67% of the 1RM with attentional focus can activate muscles at more than 85% of the 1RM. The results confirm that exercise adopting an attentional focus can result in more muscle contractions in both isolated and compound exercises.

### 3.8. Diversity in Human Physiology

In this study, the detection of attentional focus was conducted in a practical scenario. Our system allowed us to monitor attentional focus during different 1RMs and exercises. The assistive methods currently used involve the verbal and physical gestures of the trainer or application. These methods emphasize form and maximum power when lifting heavy weights, perpetuating the myth of ‘no pain, no gain’ [19]. In contrast, our system evaluated the effectiveness of exercise through muscle contraction and attentional focus based on the differences between light- and heavy-load exercises, as shown in Figure 11. The most effective training goal can be achieved by training with an appropriate weight. It should be noted that our proposed system is only a research prototype and not a mature industrial product, which raises some concerns. The first-time user must perform under two attentional focus conditions. We labeled the data collected to establish the ground truth. Consequently, the proposed system tends to be more personalized than generalized. Muscle contractions also need to be determined because of the individual differences in muscle mass. Muscle contractions may vary among individuals, depending on their physical condition. On the one hand, the limitation of the current study is that there is no threshold value to recognize attentional focus during exercise because people perceive and experience attentional focus in different ways. However, this scope allows the user to start benefiting from systems optimized for individuals once they monitor their exercise effectiveness.

We believe that there is an interest in exploring our system in the real world for interactive sports trainers for all these reasons. The diversity of human physiology makes it difficult to conduct experiments on females and other age groups. The system is currently limited because it requires calibration per user and manually checks electrode placement. The system also requires users to test their lifting capacities in advance. Our study selected exercises based on previous studies on different muscle groups, both isolated and compound exercises. For example, pullovers and bench presses are designed to train chest muscles. Females may not be suitable for our prototype shirt, and heavy exercise may result in injury in certain age groups. Considering that women and other age groups differ physiologically from men of the age group studied here, it is necessary to exclude them from training. However, we envision that the proposed system can be used in various daily activities. Future work will include field studies with a broader focus on fatigue-inducing conditions. Collecting data from a larger pool can extend our findings.

## 4. User Study

### 4.1. User Study Design

To evaluate the user experience of our system, we asked all subjects to complete modified versions of the intrinsic motivation inventory (IMI) [32], a widely used questionnaire to evaluate the user experience as shown in Table 2. Some rejected questions that were redundant to the other questions were used to check for unwilling responses. After completing the questionnaire, semi-structured interviews were conducted. We asked the participants to talk about their overall exercise experiences and suggestions.

### 4.2. User Study Results

**Figure 12:**
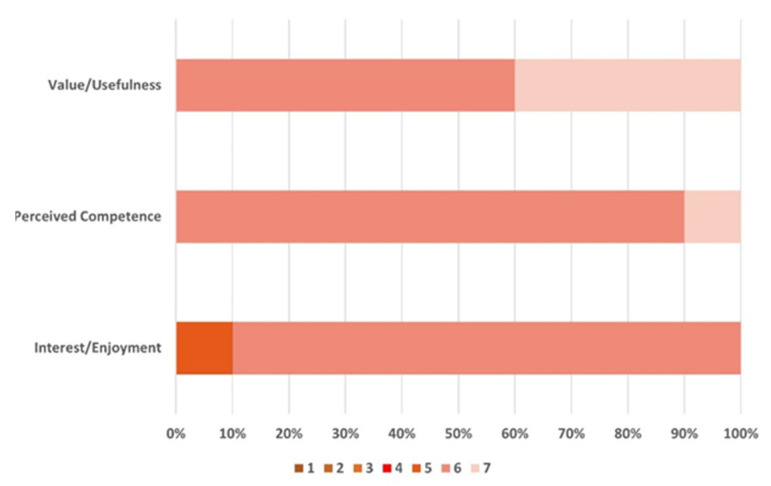
Response to the IMI questionnaire regarding interest/enjoyment, perceived competence, and value/usefulness of our system.

In the IMI, responses are graded on a seven-point Likert scale divided into seven subscales. Three subscales are relevant to our application: interest/enjoyment, perceived competence, and value/usefulness. All questions were randomly assigned to categories and averaged for statistical analysis. Figure 12 summarizes the responses to the IMI questionnaire. Participants were satisfied with the value/usefulness (M = 6.50, SD = 0.58), and four participants (40%) expressed significant agreement. Furthermore, most participants showed a positive response to perceived system competence (M = 6.08, SD = 0.61) and interest/enjoyment (M = 5.87, SD = 0.70). Following the feedback from the questionnaire, we conducted an in-depth interview. We questioned the subjects about their experiences with the system and the new information they gained.

User encounters. In general, all participants were enthusiastic about the system: “(P6) Compared to other similar products on the market, the biggest difference of the system is that it can be worn on the body and accurately detect the muscle activity of a specific part.” and “(P1) It also helps me focus on my muscle while doing exercise.” Almost everyone mentioned that the other products they used could not provide helpful suggestions while strengthening the muscles: “(P10) My smartwatch only detects the heartbeat, which cannot guide or detect specific muscle activation.”

Improvements and suggestions. Participants provided improvements and suggestions for the system. Although the equipment was very portable, “(P3) it felt obvious pressure when worn during reuse” and “(P4) it made me feel uncomfortable after being used for a long time.” Furthermore, one of the participants “(P3) felt a little afraid of touching the equipment during the experiment.”

Interactions. The participants enjoyed the effective interaction that helped them recognize muscle contraction and track the progress of the exercise: “(P7) It definitely helped me feel and focus on my muscle when I workout.”; “(P6) it can intuitively and easily reflect whether my muscle force is correct and whether I can borrow it.” Many participants agreed that it was beneficial to let them focus on the exercise and provide helpful feedback. (P2) mentioned that “there were not only some incomprehensible data.” After the experiment, half of the participants showed great interest in the system and spent more time exercising. In addition, they all want to recommend the system to their family and friends who regularly participate in fitness.

## 5. Conclusions

In this paper, we present a system that utilizes a multitasking and multiclassification network and a wearable EMG sensing device to monitor attentional focus with a robust signal quality. The purpose of our study was to develop a noninvasive, eight-channel EMG fitness shirt for the detection of attentional focus during exercise. Using a multitask and multiclassification network, we developed a system for tracking personal fitness at the muscle level by detecting attentional focus on muscle contractions from EMG signals. As a result of the implementation and evaluation of the system, we were able to obtain information regarding attentional focus and muscle contraction for a variety of lifted weights based on five standard exercises for isolated and compound muscles. Our study suggests that the system can classify the user’s attentional focus by analyzing the user’s training stage via the EMG signals obtained with the proposed device, with an average precision of approximately 94.79% among subjects at various maximum forces. In addition, the proposed system can recognize 67% and 85% of the 1RM to investigate the effectiveness of muscle activation in each session among the subjects. In addition, the findings show that low-intensity exercise at 67% of the 1RM can increase upper-limb muscle contraction more than at 85% of the 1RM.

## Figures and Tables

**Table 1:** Recognition of exercise types.

Exercise	Bench Press	Pullover	Front Raise	Kick Back	Biceps Curl
precision (%)	98.61	99.31	98.95	99.65	98.96
recall (%)	98.26	100	97.92	99.31	100

**Table 2:** User experience questionnaires.

No.	Questions	Subscale
1	I think this system could help me to know the effectiveness of training.	Value/Usefulness
2	I think I am pretty good at doing exercises with this system.	Perceived Competence
3	I would describe this system as very interesting.	Interest/Enjoyment
4	I thought using this system was quite pleasant.	Interest/Enjoyment
5	I think this system is beneficial for exercise.	Value/Usefulness
6	I think this system could do very well to monitor whether exercise is effective.	Perceived Competence
7	I was satisfied with this system when I exercised for this task.	Perceived Competence
8	While exercising with this system, I thought about how much I enjoyed it.	Interest/Enjoyment
9	Performing exercise with this system didn’t occupy my attention at all.	Interest/Enjoyment
10	I believe that using this system could be beneficial to me.	Value/Usefulness
11	Using this system for exercise was fun.	Interest/Enjoyment
12	I think this system is important because it can tell whether the exercise is effective	Value/Usefulness
13	After using this system to exercise for a while, I felt it was good.	Perceived Competence
14	I think I did well with this exercise system compared to other auxiliary devices.	Perceived Competence
15	I think this system could be of some value to me.	Value/Usefulness
16	I felt that this system was useful to monitor whether exercise was effective.	Perceived Competence
17	I enjoyed using this system.	Interest/Enjoyment
18	I would be willing to use this system again because it has some value.	Value/Usefulness
19	I think this system is a necessary tool for exercise.	Value/Usefulness
20	I thought it was not boring to use this system to do exercise	Interest/Enjoyment

## Data Availability

Not applicable.
